# Maternal age is associated with apoptotic gene abundance patterns in blastocoel fluid-conditioned media from euploid embryos: a pilot study

**DOI:** 10.1007/s10815-025-03485-7

**Published:** 2025-04-22

**Authors:** Dieu Thao Nguyen, Hannah Archer, Angel Earle, Eleanor Petyak, Carson Collins, Kayla Vaillant, Hayes Lanford, William E. Roudebush, T. Arthur Chang, Rich Kordus, Lisa Green, Alyssa Clay-Gilmour, Renee J. Chosed

**Affiliations:** 1 Department of Biomedical Sciences, University of South Carolina School of Medicine Greenville, 607 Grove Rd., Greenville, SC 29605 USA; 2 Department of Epidemiology and Biostatistics, University of South Carolina Arnold School of Public Health, Columbia, SC USA; 3 Department of Obstetrics and Gynecology, University of Texas Health Science Center, San Antonio, TX USA; 4 Department of Obstetrics and Gynecology, Prisma Health, Greenville, SC USA

**Keywords:** Apoptosis, In vitro fertilization, Advanced maternal age, CASP8, SHARPIN, BCL2L12

## Abstract

**Purpose:**

This retrospective study measured global gene abundance using RNASeq of blastocoel fluid-conditioned media from euploid ICSI-generated embryos to identify genes and signaling pathways associated with maternal age.

**Methods:**

Blastocoel fluid-conditioned media was obtained following trophectoderm biopsy of ICSI-generated day-5 blastocysts. Media for RNASeq were from 24 euploid blastocysts (9 from patients aged 35 or older). Transcriptome analysis identified differentially expressed genes when comparing media from patients of advanced maternal age to those younger than 35. Further gene abundance analysis on genes and pathways identified from the RNASeq analysis was conducted with another group of media samples using RT-qPCR.

**Results:**

Twenty-five protein encoding genes identified in the RNASeq study were differentially expressed when comparing blastocoel fluid-conditioned media associated with patients of advanced maternal age to media associated with patients under the age of 35. Genes encoding the proteins SHARPIN and BCL2L12 showed a statistically significant increase (*p* < 0.05) in abundance in patients of advanced maternal age. Abundance analysis using RT-qPCR in additional media samples revealed elevated *SHARPIN* abundance in media associated with successful implantation in patients under 35 alongside a decrease in *CASP8* abundance. This abundance pattern was the opposite in media associated with successful implantation in patients of advanced maternal age.

**Conclusions:**

This study uncovered differential apoptotic gene abundance associated with maternal age by assessing blastocoel fluid-conditioned media from euploid blastocysts. These unique abundance patterns may provide insight into the regulation of apoptosis in embryos from women of advanced maternal age, and how this signaling pathway may impact implantation outcomes.

**Supplementary Information:**

The online version contains supplementary material available at 10.1007/s10815-025-03485-7.

## Introduction

Advanced maternal age (AMA), defined as being 35 years or older, is associated with a marked decline in fecundity and an increased risk of spontaneous abortions and aneuploidy, impacting fertility outcomes [1]. The birth rate in the USA among females aged 35–44 in 2022 was observed to have increased compared to the previous 2 years [2]. With more females deciding to delay having children until later in life, they are increasingly turning to assisted reproductive technologies (ART), particularly in vitro fertilization (IVF), to address age-related infertility (reviewed by [3]).

IVF provides patients and families who are experiencing infertility with the opportunity to have a biological child. Despite continuous advancements in reproductive endocrinology, the rate of live birth outcomes with IVF has remained between 50 and 65%, a rate which decreases with age. In 2022, the National Summary Report from the Society for Assisted Reproductive Technology (SART) reported a total of 389,993 IVF cycles, of which approximately 60% of the patients ≥ 35 years old. Patients < 35 years old who utilized IVF had a successful live birth outcome rate of 43.1%. Patients aged 35–37 had a success rate of 31.0%, but for patients above 40 years old, the chances of live birth outcomes decreased to a rate of between 3.2 and 19.0% [4].

Increase in maternal age has been associated with a decline in oocyte quantity and quality. There is a finite number of oocytes, around 1–2 million, after birth. Throughout the reproductive life cycle, the finite amount dwindles to approximately a few hundred in the perimenopausal period. Oocyte quality declines through a variety of proposed mechanisms including the regulating process, mitochondria competence, spindle assembly, and chromosomal nondisjunction, all potential contributers to increasing rates of aneuploidy associated with maternal age [5].

AMA patients have a significant increase in embryo aneuploidy rate, from 25 to 30% between the ages of 28 and 29 to 90% in their late 40 s prior to menopause [3, 6]. Chromosomal abnormalities are strongly associated with first trimester pregnancy loss [6]. Efforts to improve live birth outcomes include preimplantation genetic testing for aneuploidy (PGT-A), which has been employed to screen for the most viable embryo for transfer. PGT-A involves a biopsy of the trophectoderm cells (TE) from day 5/6 IVF embryos and subsequent next generation sequencing of the DNA to detect any chromosomal abnormalities. The utility of PGT-A has been debated as the presence of mosaicism within an embryo presents a potential limiting factor to the accuracy of this screening tool [7]. Mosaicism is a phenomenon in which there are chromosomally distinct cells within an individual; thus, TE cells that are biopsied for this test may not accurately capture ploidy status of the inner cell mass (ICM) of the embryo. However, Sachdev et al. reported that the concordances between TE cells and ICM are 99.5%, 97.3%, and 35.2% for euploid, aneuploid, and mosaic embryos, respectively [8].

A multinational and multicenter randomized control trial revealed that PGT-A screening did not improve ongoing pregnancy at 20-week gestation for patients < 35, but there was a statistically significant increase in ongoing pregnancy for patients 35–40 years old [9]. Additionally, a recent study involving the randomization of 1212 patients into two groups, conventional IVF and IVF with additional PGT-A screening, revealed that conventional IVF is noninferior to PGT-A [10]. While PGT-A has shown benefits for embryo selection for AMA patients and those who have a history of recurrent pregnancy loss, there are compelling arguments against its overall efficacy. To improve embryo selection, additional and more precise biomarkers of IVF embryo viability need to be identified.

Noninvasive or minimally invasive PGT-A has been investigated as a potential alternative to traditional PGT-A or as an additional screening tool [11]. This screening tool has been used to assess cell-free DNA within spent culture media from IVF embryos [12], blastocoel fluid from within the developing embryo [13] or blastocoel fluid-conditioned media. Questions remain about the quality and the origin of the DNA being assessed in the spent media and how comparable this source of DNA is to that obtained from the trophectoderm [11]. Additional research has assessed the metabolome in spent media from IVF blastocysts to identify markers of embryo competence [14]. The spent culture media has also been used to assess microRNA content as well as gene expression. miR- 294 was detected in media obtained from IVF blastocysts, and interestingly, this microRNA is associated with apoptosis [15]. Similarly, apoptotic gene expression was detected in blastocoel fluid-conditioned media [16].

The detection of apoptotic factors or remnants in culture media should be anticipated given that apoptosis occurs during early embryo development [17, 18]. The role of apoptosis in eliminating aneuploid or damaged cells during embryo development has been intensely studied as this process may impact embryo viability [19, 20]. Mitochondria are crucial for the process of apoptosis, and mitochondria in an embryo are received from the oocyte. Given that oocyte aging is associated with changes in mitochondria morphology, reduced mtDNA copy number, mtDNA mutations, and reduced mitochondrial function, it is likely that the developing embryo from patients of advanced maternal age may have reduced viability due to its inherited mitochondria [21].

This study assessed global gene abundance using RNA-Seq in blastocoel fluid-conditioned media from euploid ICSI-generated embryos from patients of varying maternal age. Gene abundance likely varies with patient age and embryo implantation outcome. Therefore, this study aimed to identify genes and associated pathways linked with maternal age.

## Materials and methods

### Blastocoel fluid-conditioned media collection

Blastocoel fluid-conditioned media were collected at Fertility Center of the Carolinas (Prisma Health, Greenville, SC) and the UT Health San Antonio (San Antonio, TX) following standard procedure for IVF with intracytoplasmic sperm injection (ICSI) and embryo biopsy for preimplantation genetic testing for aneuploidies (PGT-A). Additional informed consent was obtained from all patients at Fertility Center of the Carolinas as per the Prisma Health IRB. Following 5–6 days of embryo culture, the blastocyst-stage embryos were transferred into a fresh drop (25 μL) of culture medium in preparation for embryo biopsy. Laser pulses were applied between the cellular junctions of the extruded trophectoderm (TE) cells and cells within the zona pellucida to allow removal of these cells for further analysis. PGT-A of the biopsied TE cells was performed via Next-Gen Sequencing (NGS) at a commercial sequencing company. Upon conclusion of the TE cell biopsy, the blastocyst extruded blastocoel fluid (~ 5 nL in volume) through the gaps formed into the surrounding biopsy medium. The blastocoel fluid-conditioned media were collected and saved post biopsy. The blastocoel fluid-conditioned media were snap frozen at − 20 °C and then stored at − 80 °C prior to analysis. The biopsied embryos were cryopreserved (liquid nitrogen) pending outcome of the NGS. All embryos used in this study were subjected to PGT-A analysis (CooperGenomics); thus, they were frozen prior to transfer. No media was obtained from thawed/re-biopsied embryos. All embryos used in this study were transferred within 2 years of embryo cryopreservation with the majority being transferred in less than 1 year. No embryos derived from donor oocytes were used in this study.

### RNASeq

**Fig. 1:**
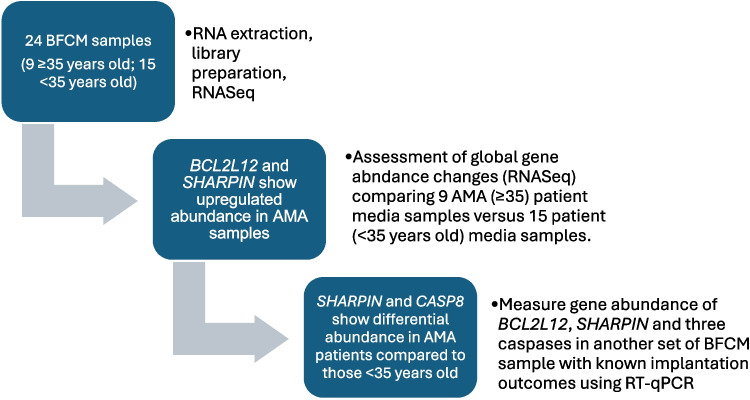
Methods flowchart

Blastocoel fluid-conditioned media from 12 embryos that resulted in successful implantation and 12 embryos resulting in unsuccessful implantation were selected for RNASeq analysis (Table 1). These samples represented patients ranging from 26 to 41 years of age, nine samples of which were from patients of advanced maternal age. RNA extraction, library preparation, sequencing, and post-processing of the raw data were conducted at the University of South Carolina CTT COBRE Functional Genomics Core (Fig. 1). RNA from the media was extracted via the Zymo Quick-RNA Microprep kit as per the manufacturer’s recommendations (Zymo Research, Irvine, CA, USA). RNA libraries were prepared using an established protocol with SMART-Seq® Stranded Kit (Takara Bio USA, Mount View, CA). Each library was made with one of the TruSeq barcode index sequences, and the Illumina sequencing was performed by FGC with Illumina NextSeq500 (75 bp, pair-ended). Raw sequencing reads (75-bp single end reads) were quality checked for potential sequencing issues and contaminants using MultiQC v1.7 [22], which includes featureCounts [23], STAR [24], Cutadapt [25], and FastQC [26]. Adapter sequences and primers were trimmed from the sequencing reads using fastQC [27], then followed by removing polyN and read portions with low complexity and/or quality score below 28 using Trimmomatic [28] and PRINSEQ [29]. Reads with a remaining length of fewer than 16 bp after trimming were discarded. Trimmed reads were mapped to the human genome (GRCh37/hg19) using bowtie [30]. Read coverage on forward and reverse strands for genome browser [31] visualization was computed using SAMtools [32], BEDtools [33], and UCSC Genome Browser utilities [31]. Raw read counts were calculated for known gene categories including ncRNAs, antisense transcripts, coding and intronic regions of mRNAs, and repeats. Annotations of known genes were retrieved from miRBase release 22 [34], NCBI RefSeq [35], Human lincRNA Catalog [36], and UCSC Genome Browser [31].

**Table 1:**
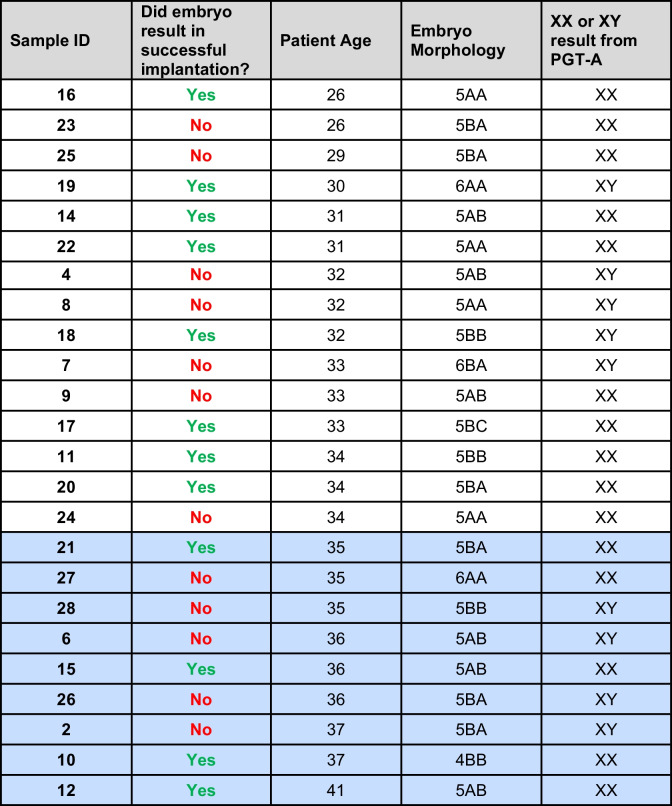
Blastocoel fluid-conditioned media sample information

Patient age listed is for the mother at the time of embryo cryopreservation. Yes or No represents whether a successful pregnancy occurred after transfer of the embryo. Chromosomal status was determined by PGT-A. Blue shaded boxes indicate media collected from embryos associated with mothers of advanced maternal age (≥ 35 years of age)

Note that a blank biopsy media sample (MHM, Irvine Scientific) was processed alongside the blastocoel fluid-conditioned media samples, and the amount of total RNA purified from the blank biopsy media sample was comparable to the negative control used with Zymo Quick-RNA Microprep kit. Therefore, the RNA purified from blank biopsy media was not used for further sequencing due to the extremely low amount of RNA.

### Statistical analysis

After quality control, the total gene count was 56,269. DESeq2, a method for differential analysis of count data, using shrinkage estimation for dispersions and fold changes to improve stability and interpretability of estimate was used for differential gene abundance analysis [37]. Raw read counts were normalized across all samples. Due to the limited number of samples and variations in the quality of read counts, all RNA types were included in the analysis. Pre-filtering was done, retaining only those rows (genes) with >  = 10 Counts (*N* = 24,347). The r-log transformation was the most effective at stabilizing the variance across the mean log2 fold change considering “pregnant vs. no pregnancy (reference).” The samples were further stratified by age; “under 35 years of age (reference) vs. age 35 and higher.” Wald test statistics were used to compare the groups. Results (adjusted FDR *p*-value < 0.05) adjusted false-discovery rate (FDR) *p*-value < 0.05 were considered statistically significant for the differential expressed genes. Normalized counts were further used in Gene set enrichment analysis (GSEA) and KEGG pathway analyses for functional annotation [38, 39].

### Gene selection process

The function of the protein products of the differentially expressed genes (*p*-value < 0.05) identified in the RNASeq study was found using the Panther database (https://pantherdb.org/). At least eight protein products were involved in apoptotic processes or regulation of apoptotic genes: *BCL2L12*, *SHARPIN*, *SIK3*, *CYB5R2*, *POU4 F1*, *KIF18 A*, *NLRP4*, and *CUL2*. Therefore RT-qPCR studies included the measurement of gene abundance of three caspase genes (*CASP3*, *CASP7*, and *CASP8*) as they play a central role in apoptosis.

### RT-qPCR

Additional blastocoel fluid conditioned media samples (166 samples) were divided into four categories: (1) mother’s age ≥ 35; successful embryo implantation (2) mother’s age ≥ 35; unsuccessful embryo implantation (3) mother’s age < 35; successful embryo implantation (4) mother’s age < 35; unsuccessful embryo implantation. Media samples per category were identified. For each category of samples, four subgroups of 7–10 media samples were formed. Finally, the media samples in each subgroup were pooled into single microfuge tubes by pipetting (four groups of pooled media samples per category). Refer to Supplentary Table 1 for media sample information. Real-time-quantitative polymerase chain reaction (RT-qPCR) was performed for each pooled sample group. Prior to RT-qPCR, RNA was extracted using a Zymo Quick-RNA Microprep kit as per the manufacturer’s recommendations. RNA samples were then subjected to cDNA synthesis (High-Capacity cDNA Reverse Transcription Kit, Applied Biosystems, USA) as per the manufacturer’s instructions. Approximatley 40 ng of cDNA (from each pooled sample group) was combined with 2X TaqMan Master Mix, 20X Gene Expression Assay (*18S*, *BCL2L12*, *SHARPIN*, *CASP3*, *CASP7*, or *CASP8* specific) and nuclease free water as per the manufacturer’s instructions (Applied Biosystems). Samples were run for each gene of interest using a 7500 Fast Real-Time PCR System (Applied Biosystems, USA) at 50 °C for 2 min, 95 °C for 20 s, followed by 40 cycles of 95 °C for 3 s and 60 °C for 30 s.

The raw *C*_*t*_ values for each subgroup were recorded. Note that a *C*_*t*_ value of 40 was assigned for samples where no abundance was detected. Δ*C*_*t*_ values (calculated for each gene of interest *C*_*t*_ value normalized against 18S *C*_*t*_) were then determined. Δ*C*_t_ values were then averaged for each subgroup. Standard error was then calculated in Excel. Refer to Supplentary Table 2 for raw values. Bar graph (Fig. 3) shows Δ*C*_*t*_ values with standard error. For further statistical analysis, Δ*C*_*t*_ values were compared using a *t*-test with SigmaPlot (SysStat Software, Inc., San Jose, CA).

**Table 2:**
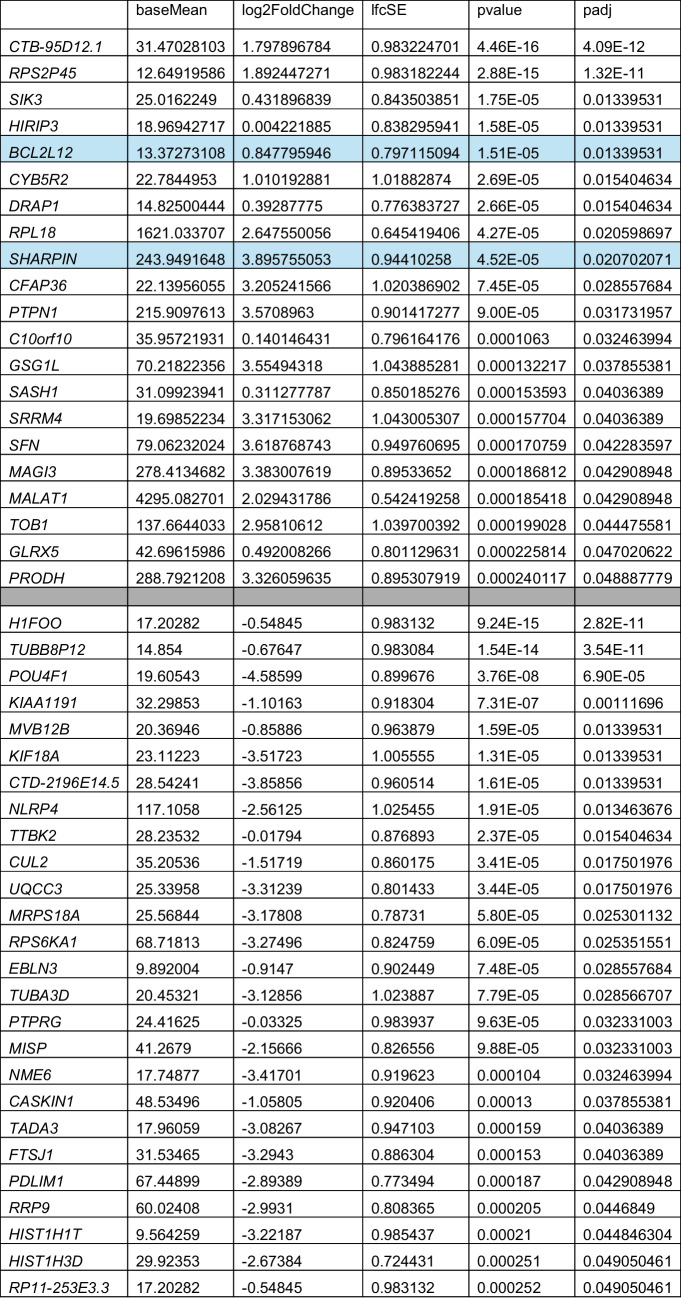
Differential gene abundance results by age (< 35 years old versus ≥ 35 years old)

*baseMean*, mean value (base); *log2 FoldChange*, log fold change; *lfcSE*, log fold change standard error; *pvalue*, unadjusted; *padj*, adjusted FDR *p*-value < 0.05

Note that a blank biopsy media sample was also included in the RT-qPCR part of the study using the same process as described abobe for the conditioned media samples. The only detectable amount of any gene present was *18S* which was comparable to that seen in the blastocoel fluid-conditioned media samples.

## Results

**Fig. 2:**
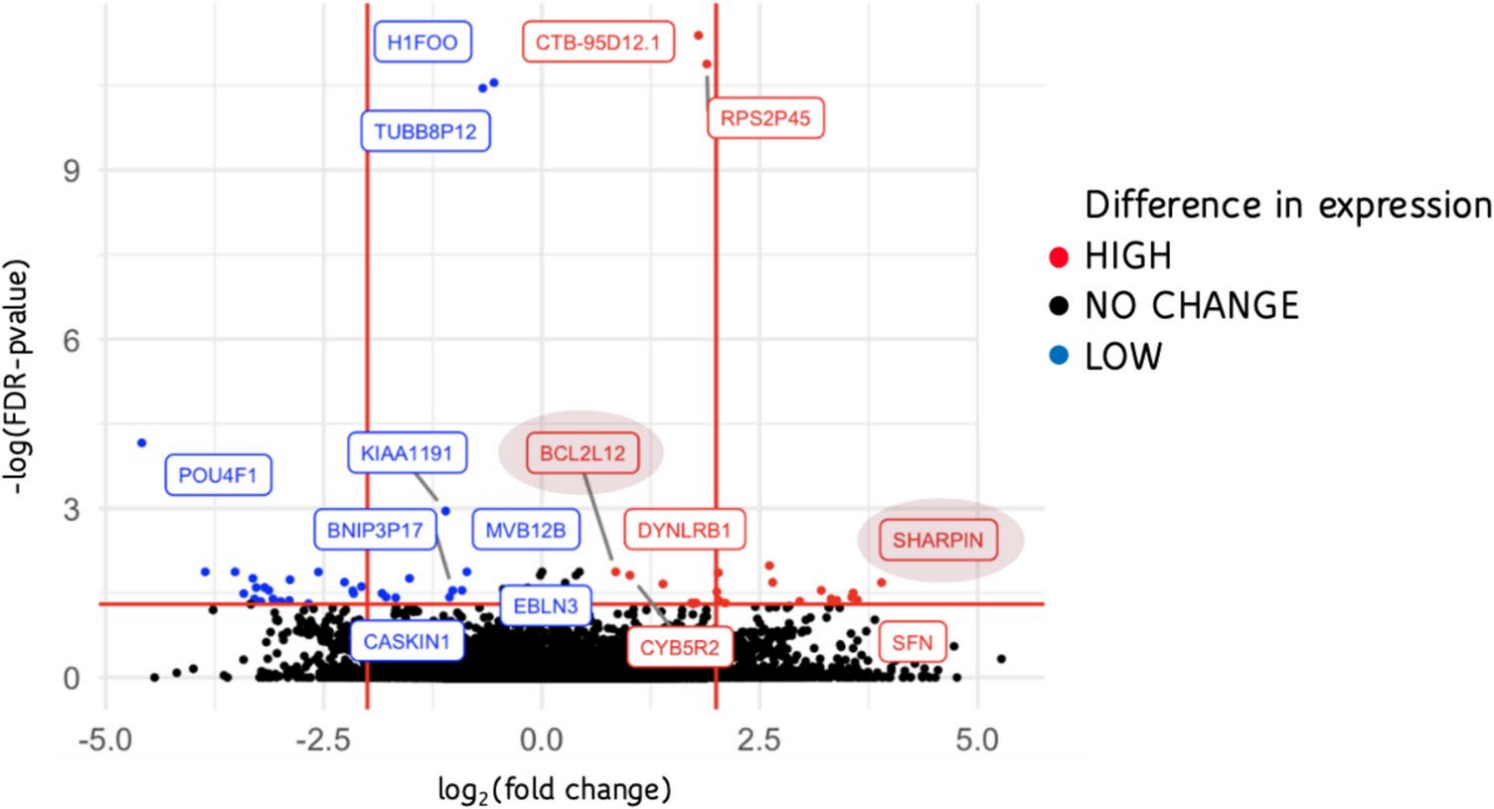
Volcano plot of differential gene abundance results by age (< 35 years old versus ≥ 35 years old)

RNASeq was performed on individual blastocoel fluid-conditioned media samples from 24 euploid embryos with known implantation status (Table 1). Forty-seven protein encoding genes identified in the RNASeq study were differentially expressed (*p* < 0.05) when comparing blastocoel fluid-conditioned media associated with patients of advanced maternal age to patients under the age of 35 (Table 2). Genes encoding the proteins SHARPIN and BCL2L12 showed a statistically significant increase in abundance in patients of advanced maternal age (Figs. 1 and 2). Both SHARPIN and BCL2L12 play a role in apoptosis as well as the protein products of six other differentially expressed genes from the RNASeq analysis (*SIK3*, *CYB5R2*, *POU4 F1*, *KIF18 A*, *NLRP4*, and *CUL2).* Therefore, RT-qPCR studies included the measurement of gene abundance of three caspase genes as their protein products play a central role in apoptosis (*CASP3*, *CASP7*, and *CASP8*).

**Fig. 3:**
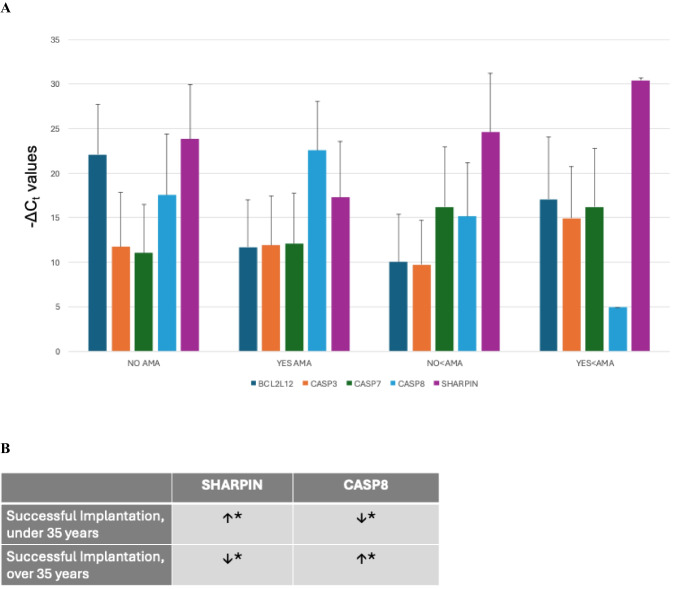
Abundance of *BCL2L12*, *CASP3*, *CASP7*, *CASP8*, and *SHARPIN* in pooled blastocoel fluid-conditioned media samples. **A** RT-qPCR in additional media samples revealed elevated *SHARPIN* abundance in media associated with successful implantation in patients under 35, alongside a decrease in *CASP8* abundance (far right set of bars) as reported with Δ*C*_*t*_ values (compared to 18S gene abundance). This abundance pattern was the opposite in media associated with successful implantation in patients of advanced maternal age (second set of bars). Error bars show standard error for each group. Labels: “No AMA” patient’s age ≥ 35, unsuccessful embryo implantation; “Yes AMA” patient’s age ≥ 35, successful embryo implantation; “No < AMA” patient’s age < 35, unsuccessful embryo implantation; “Yes < AMA” patient’s age < 35; successful embryo implantation. **B** Table of *SHARPIN* and *CASP8* gene abundance. * indicates statistical significance as follows: Difference in abundance of *SHARPIN* (*p* = 0.029) and *CASP8* (*p* = 0.029) was statistically significant when comparing AMA patients with successful implantation versus AMA patients with unsuccessful implantation

Gene abundance analysis using RT-qPCR in additional pooled media samples revealed elevated *SHARPIN* abundance in media associated with successful implantation in patients under 35, concomitant with a decrease in *CASP8* abundance (Fig. 3). This abundance pattern was the opposite in media associated with successful implantation in patients of advanced maternal age (compare the second and third groups in Fig. 3). This difference in abundance with *SHARPIN* (*p* = 0.029) and CASP8 (*p* = 0.029) was statistically significant when comparing these two groups of samples (AMA patients with successful implantation versus AMA patients with unsuccessful implantation). Increased *CASP8* abundance was also observed in media from embryos from AMA patients regardless of implantation status (Fig. 3).

*BCL2L12* abundance was lower in media associated with successful implantation when compared to media associated with unsuccessful implantation in patients of advanced maternal age (compare the first and second groups in Fig. 3). This difference was not statistically significant.

## Discussion

This study assessed gene abundance in blastocoel fluid-conditioned media using global transcriptome analysis and revealed altered abundance patterns of apoptotic genes when comparing media from patients of advanced maternal age to those under 35 years of age (Table 2, Figs. 1 and 2). Specifically, genes encoding SHARPIN and BCL2L12 were identified. SHARPIN is a component of a multi-protein E3 ubiquitin ligase complex called LUBAC [40]. This complex catalyzes the addition of ubiquitin to protein targets involved in apoptosis, inflammation, and other pathways. In mouse studies, SHARPIN functions to regulate CASP8-induced apoptosis and serves to inhibit cell death [41]. BCL2L12 is a member of the BCL2 gene family and serves an anti-apoptotic function. BCL2L12’s anti-apoptotic role is reported to impact ovarian cancer [42]. BCL2 was also reported to play a role in recurrent pregnancy loss [43]. Identification of genes that regulate apoptosis within blastocoel fluid-conditioned media is consistent with other studies [15, 16] and the role that apoptosis plays in early embryo development [2, 18, 19, 44, 45].

To further examine the abundance patterns of these two genes as well as caspases, which play a crucial role in apoptosis, another set of blastocoel fluid-conditioned media samples were assessed. Abundance of *SHARPIN*, *BCL2L12*, *CASP3*, *CASP7*, and *CASP8* was measured in pooled media samples using RT-qPCR. This analysis revealed a unique abundance pattern of *SHARPIN* alongside *CASP8*. There was elevated *SHARPIN* abundance in media associated with successful implantation in patients under 35, concomitant with a decrease in *CASP8* abundance (Fig. 3). Interestingly, this gene abundance pattern was the exact opposite when looking at the abundance of these same two genes in media associated with successful implantation in patients of advanced maternal age (Fig. 3). Further analysis of *CASP8* abundance across the pooled media groups revealed that *CASP8* abundance was also elevated in media from patients of advanced maternal age regardless of the embryo implantation status (Fig. 3).

These results suggest that increased *CASP8* abundance is associated with advanced maternal age and negative implantation outcomes in patients under 35. CASP8 may be elevated in the developing blastocyst to trigger apoptotic cell death, more so in blastocysts from AMA patients or blastocysts that fail to implant. One potential explanation is that there are more aneuploid cells in blastocysts from patients of advanced maternal age, and thus apoptosis is occurring more frequently in these embryos as a self-correction mechanism. Previous studies have detected depletion of aneuploid cells in human and mouse mosaic and aneuploid embryos via apoptosis, but have not directly assessed the impact of age in this process [44, 46–48]. However, it is important to note that this study was limited in the use of euploid embryos that were then transferred to patients, and studies reporting self-correction (removal of aneuploid cells) used mosaic or aneuploid embryos. A study by Popovic et al. reported that extended culture of human mosaic embryos did result in many of the embryos eliminating aneuploid cells and resulting in euploid embryos [48]. The extended culture study provides rationale that human embryos classified as euploid via PGT-A may have harbored aneuploid cells at some point during early development. Since this study also showed elevated *CASP8* in media from embryos that failed implantation in patients under 35, it is possible that these embryos increased apoptosis to remove aneuploid cells from the embryo, but potentially did not succeed in sufficient removal to allow for implantation. These results may indicate that blastocysts with less of a need for self-correction (potentially fewer aneuploid cells) have lower abundance of CASP*8*. Therefore, lower abundance of CASP8 could be an additional indicator to select which euploid embryo may have the best potential for successful implantation. CASP8 could be activated in preimplantation embryos as an aging response to promote self-correction via apoptosis in media samples from AMA patients or media from embryos that did not successfully implant.

Interestingly, SHARPIN has been reported in the literature to inhibit CASP8 and CASP9 in B-cells [49] and inhibit apoptosis in mouse keratinocytes through a mechanism that includes CASP8 [41]. While this current study does not investigate the mechanistic link between SHARPIN and CASP8, these studies offer insight into a potential mechanism of SHARPIN inhibiting CASP8 when self-correction is no longer needed in the early blastocyst. This study found a statistically significant increase in abundance of SHARPIN in media from euploid embryos from patients under 35 years of age, and it is possible that this increased abundance inhibits that of CASP8 since these embryos would be considered viable and may not need additional self-correction via apoptosis.

*BCL2L12* showed reduced abundance in media associated with successful implantation compared to unsuccessful implantation, but only in patients of advanced maternal age (Fig. 3). This difference was not statistically significant and was not observed in patients under 35 years of age. BCL2L12 is considered an anti-apoptotic protein, and it is therefore possible that its abundance was reduced in the media associated with successful implantation because these embryos did not require self-correction via apoptosis. Conversely, BCL2L12 might be activated as a stress response in embryos requiring more self-correction via apoptosis. The self-correction process may be insufficient in some embryos and potentially increase the likelihood of negative implantation outcomes.

## Supplementary Information

Below is the link to the electronic supplementary material.

Supplementary file1 (DOCX 29 KB)

Supplementary file2 (DOCX 100 KB)

## Data Availability

Data will be made available to the editors of the journal for review or query upon request.
