# Supplementary material for: Maternal age is associated with apoptotic gene abundance patterns in blastocoel fluid-conditioned media from euploid embryos: a pilot study
Source: J Assist Reprod Genet. 2025 Apr 22;42(5):1651–61. doi: 10.1007/s10815-025-03485-7 (PMC12167202; doi:10.1007/s10815-025-03485-7)
Supplement: Supplementary file 1 — Supplementary file1 (DOCX 29 KB) [file 10815_2025_3485_MOESM1_ESM.docx]

Supplementary Table 2. Blastocoel-fluid conditioned media sample information. Age listed is for the mother at the time of embryo cryopreservation. “Yes” or “No” represents the embryo implantation outcome.

| Sample # | Did embryo result in successful implantation? | Patient Age | Embryo Morphology |
| --- | --- | --- | --- |
| 1 | NO | 24 | 4AA |
| 2 | NO | 29 | 6AA |
| 3 | NO | 31 | 4BB |
| 4 | NO | 31 | 3AA |
| 5 | NO | 31 | 3BB |
| 6 | NO | 31 | 5BB |
| 7 | NO | 31 | 4AA |
| 8 | NO | 32 | 4AA |
| 9 | NO | 32 | 5AA |
| 10 | NO | 32 | 5AA |
| 11 | NO | 32 | 6BB |
| 12 | NO | 32 | 5AB |
| 13 | NO | 32 | 5CB |
| 14 | NO | 32 | 5AA |
| 15 | NO | 32 | 5BB |
| 16 | NO | 32 | 6AB |
| 17 | NO | 32 | 6AA |
| 18 | NO | 33 | 6AA |
| 19 | NO | 33 | 5AB |
| 20 | NO | 33 | 6BA |
| 21 | NO | 33 | 6AA |
| 22 | NO | 34 | 4AA |
| 23 | NO | 34 | 4AA |
| 24 | NO | 34 | 5BB |
| 25 | NO | 34 | 5AA |
| 26 | NO | 34 | 5AB |
| 27 | NO | 34 | 5BC |
| 28 | NO | 34 | 6BA |
| 29 | YES | 26 | 5AA |
| 30 | YES | 29 | 5AA |
| 31 | YES | 29 | 6AB |
| 32 | YES | 30 | 4AA |
| 33 | YES | 30 | 5CB |
| 34 | YES | 30 | 6AA |
| 35 | YES | 30 | 5BC |
| 36 | YES | 30 | 6AA |
| 37 | YES | 31 | 3AA sz 2 |
| 38 | YES | 31 | 5AB |
| 39 | YES | 31 | 6AA |
| 40 | YES | 31 | 6AA |
| 41 | YES | 31 | 4AA |
| 42 | YES | 31 | 5AB |
| 43 | YES | 31 | 5AA |
| 44 | YES | 31 | 5AB |
| 45 | YES | 32 | 5AB |
| 46 | YES | 32 | 4AB |
| 47 | YES | 32 | 5AA, size 3 |
| 48 | YES | 32 | 4AA |
| 49 | YES | 32 | 5BA |
| 50 | YES | 32 | 6AA |
| 51 | YES | 32 | 5BA |
| 52 | YES | 32 | 5BB |
| 53 | YES | 32 | 5BA |
| 54 | YES | 33 | 5BC |
| 55 | YES | 33 | 5BA |
| 56 | YES | 34 | 5AA |
| 57 | YES | 34 | 5AB |
| 58 | YES | 34 | 5AB |
| 59 | YES | 34 | 5BB |
| 60 | YES | 34 | 5BA |
| 61 | YES | 34 | 5BB |
| 62 | NO | 35 | 5AA |
| 63 | NO | 40 | 3AA |
| 64 | NO | 40 | 3BC |
| 65 | NO | 35 | 4BC |
| 66 | NO | 35 | 4CC |
| 67 | NO | 35 | 6AA |
| 68 | NO | 49 | 4AA |
| 69 | NO | 49 | 4BB |
| 70 | NO | 49 | 3BC |
| 71 | NO | 49 | 3BC |
| 72 | NO | 49 | 3AC |
| 73 | NO | 37 | 3BB |
| 74 | NO | 42 | 5AA |
| 75 | NO | 42 | 6AB |
| 76 | NO | 39 | 4AB |
| 77 | NO | 39 | 4CB |
| 78 | NO | 36 | 6AA |
| 79 | NO | 40 | 4AA |
| 80 | NO | 40 | 4BB |
| 81 | NO | 40 | 3BC |
| 82 | NO | 40 | 4AC |
| 83 | NO | 40 | 3BC |
| 84 | NO | 41 | 5AB |
| 85 | NO | 36 | 4AA |
| 86 | NO | 39 | 5BB |
| 87 | NO | 37 | 4AA |
| 88 | NO | 37 | 4BB |
| 89 | NO | 37 | 4AA |
| 90 | NO | 37 | 4AA |
| 91 | NO | 42 | 5BA |
| 92 | NO | 38 | 4AC |
| 93 | NO | 38 | 4CB |
| 94 | NO | 38 | 3AA |
| 95 | NO | 43 | 4AA |
| 96 | NO | 40 | 4AA |
| 97 | NO | 35 | 4AA |
| 98 | NO | 36 | 5AB |
| 99 | NO | 39 | 5BB (size 3) |
| 100 | NO | 36 | 3AA |
| 101 | NO | 36 | 3CB |
| 102 | NO | 36 | 4AA |
| 103 | NO | 39 | 4AA |
| 104 | NO | 38 | 3AC |
| 105 | NO | 38 | 5AB |
| 106 | NO | 36 | 6AA |
| 107 | YES | 35 | 5AA |
| 108 | YES | 35 | 3AB |
| 109 | YES | 35 | 5AA (size 3) |
| 110 | YES | 35 | 4AA |
| 111 | YES | 35 | 5AA |
| 112 | YES | 36 | 6AB |
| 113 | YES | 36 | 5AB |
| 114 | YES | 36 | 5BB |
| 115 | YES | 36 | 5AA |
| 116 | YES | 36 | 4AA |
| 117 | YES | 36 | 5AA |
| 118 | YES | 36 | 4AA |
| 119 | YES | 36 | 5AB (size 3) |
| 120 | YES | 36 | 5AA |
| 121 | YES | 36 | 5AB |
| 122 | YES | 36 | 5AA |
| 123 | YES | 37 | 6AA |
| 124 | YES | 37 | 4AA |
| 125 | YES | 37 | 4CB |
| 126 | YES | 37 | 4AA |
| 127 | YES | 37 | 3CB |
| 128 | YES | 37 | 3BB |
| 129 | YES | 37 | 4BB |
| 130 | YES | 37 | 4AA |
| 131 | YES | 38 | 5AB (size 3) |
| 132 | YES | 38 | 4AC |
| 133 | YES | 38 | 5AA (size 3) |
| 134 | YES | 38 | 4AA |
| 135 | YES | 38 | 4AA |
| 136 | YES | 38 | 4CB |
| 137 | YES | 38 | 4AA |
| 138 | YES | 38 | 4AA |
| 139 | YES | 39 | 5BA (size 3) |
| 140 | YES | 39 | 6AA |
| 141 | YES | 39 | 4AA |
| 142 | YES | 39 | 6CC |
| 143 | YES | 39 | 4BA |
| 144 | YES | 39 | 3CC |
| 145 | YES | 39 | 4AA |
| 146 | YES | 39 | 5BB |
| 147 | YES | 39 | 5AA |
| 148 | YES | 40 | 4AA |
| 149 | YES | 40 | 5AA |
| 150 | YES | 40 | 5AA |
| 151 | YES | 40 | 5AA |
| 152 | YES | 40 | 5AA |
| 153 | YES | 41 | 5AA |
| 154 | YES | 41 | 4AA |
| 155 | YES | 41 | 4AA |
| 156 | YES | 41 | 5AB |
| 157 | YES | 41 | 5CC |
| 158 | YES | 41 | 5AA |
| 159 | YES | 41 | 5BB (size 3) |
| 160 | YES | 41 | 5AA |
| 161 | YES | 42 | 3AA |
| 162 | YES | 42 | 4AA |
| 163 | YES | 42 | 4AA |
| 164 | YES | 42 | 5AB |
| 165 | YES | 43 | 4AA |
| 166 | YES | 43 | 4AA |
